# Oxytocin alters cell fate selection of rat neural progenitor cells *in vitro*

**DOI:** 10.1371/journal.pone.0191160

**Published:** 2018-01-18

**Authors:** Arvind Palanisamy, Ramaswamy Kannappan, Zhiqiang Xu, Audrey Martino, Matthew B. Friese, Justin D. Boyd, Gregory Crosby, Deborah J. Culley

**Affiliations:** 1 Department of Anesthesiology, Perioperative and Pain Medicine, Brigham and Women’s Hospital, Harvard Medical School, Boston, Massachusetts, United States of America; 2 Department of Anesthesiology, Washington University School of Medicine, St. Louis, Missouri, United States of America; 3 Senior Manager of Biology, Beryllium Discovery, Bedford, MA, United States of America; University of Pennsylvania, UNITED STATES

## Abstract

Synthetic oxytocin (sOT) is widely used during labor, yet little is known about its effects on fetal brain development despite evidence that it reaches the fetal circulation. Here, we tested the hypothesis that sOT would affect early neurodevelopment by investigating its effects on neural progenitor cells (NPC) from embryonic day 14 rat pups. NPCs expressed the oxytocin receptor (OXTR), which was downregulated by 45% upon prolonged treatment with sOT. Next, we examined the effects of sOT on NPC death, apoptosis, proliferation, and differentiation using antibodies to NeuN (neurons), Olig2 (oligodendrocytes), and GFAP (astrocytes). Treated NPCs were analysed with unbiased high-throughput immunocytochemistry. Neither 6 nor 24 h exposure to 100 pM or 100 nM sOT had an effect on viability as assessed by PI or CC-3 immunocytochemistry. Similarly, sOT had negligible effect on NPC proliferation, except that the overall rate of NPC proliferation was higher in the 24 h compared to the 6 h group regardless of sOT exposure. The most significant finding was that sOT exposure caused NPCs to select a predominantly neuronal lineage, along with a concomitant decrease in glial cells. Collectively, our data suggest that perinatal exposure to sOT can have neurodevelopmental consequences for the fetus, and support the need for in vivo anatomical and behavioral studies in offspring exposed to sOT *in utero*.

## Introduction

Synthetic oxytocin (sOT), marketed in the United States as Pitocin^**®**^, is widely used for either induction and/or augmentation of labor, and to prevent postpartum haemorrhage. Between 1998 and 2007, the incidence of induction of labor alone more than doubled from 9.8% to 23%.[1] The continuous administration of supraphysiological doses of sOT for either induction or augmentation of labor is strikingly different from the pulsatile pattern of endogenous oxytocin release that occurs during unmedicated delivery.[2] Though the mechanical effects of sOT on the uterus are well-studied and it is clear that maternally administered sOT reaches the fetal circulation in humans,[3] little is known about whether it affects fetal neurodevelopment. There is, however, reason to believe it may. In situ hybridization histochemistry reveals that oxytocin receptor (OXTR) mRNA is present in rat brain as early as embryonic day 13 (E13)[4] and binding studies show OXTRs in mouse brain by E18.5.[5] Likewise, binding studies show OXTRs in the posterior dorsal neural tube of fetal rats at E14 [6] and on cultured astroglial cells isolated from E16 rat fetuses.[7] Furthermore, there is transcriptomic evidence for OXTR expression in the developing second trimester human brain (Human Brain Transcriptome Project).[8, 9] Finally, epidemiological evidence suggests an association between labor induction practices such as oxytocin and the risk of neurodevelopmental disorders.[10–14] Though causality has not been definitively established and there are more induced labors than children with neurodevelopmental disorders, it is plausible that sOT is one of several environmental factors that trigger or unmask underlying genetic susceptibility to neurodevelopmental disorders.[15] However, its contribution is unclear because the impact of maternally administered sOT on neurodevelopmental events in the fetus has not been systematically examined.

Neural stem/progenitor cells (NPCs) are critical for normal early brain development. NPCs differentiate into most cell types in the brain including neurons, astrocytes, and oligodendrocytes[16–18] and abnormalities in NPC biology are implicated in neurodevelopmental disorders.[19] Proliferation and differentiation of NPCs are genetically programmed, yet these cells are highly sensitive to environmental, pharmacological, and cerebrospinal fluid (CSF)—guided cues.[20] This is relevant here because NPCs in neurogenic niches are continually bathed in CSF and peripherally administered sOT enters the CSF within 10 min and has a half-life in CSF that is 6-fold longer than in plasma.[21, 22] In addition, sOT influences proliferation and differentiation of multiple cell lines in vitro and in vivo.[23–30] Based on this information, we hypothesize that NPCs may be a target of sOT and that exposure to oxytocin alters both proliferation and differentiation of these cells. To test this hypothesis, we first determined if NPCs express the OXTR, subsequently investigated the effect of prolonged treatment with sOT on OXTR protein expression in NPCs, and finally evaluated the effect of prolonged sOT treatment on the viability, proliferation, and differentiation of NPCs.

## Materials and methods

All animal experiments were approved by IACUC and conducted according to regulations set forth by the Harvard Medical Area Standing Committee on Animals (Boston, MA).

### NPC culture

Neural stem/progenitor cells were harvested from timed pregnant embryonic day 14 Sprague Dawley rats (Harlan Sprague Dawley, Indianapolis, IN) as previously described.[31, 32] Briefly, embryonic NPCs were isolated from the telencephalon of unborn fetuses of 26 Sprague Dawley rats on day 14 of gestation (E14) after 100% CO_2_ euthanasia. The harvests were done sequentially such that at any given point in time, only NPCs from that particular culture were used for experiments. NPCs were cultured in B27 medium, which consists of Dulbecco's Modified Eagle Medium/F12 high glucose (Invitrogen), supplemented with glutamine (1:200, Invitrogen), Fungizone^®^ antimycotic (1:100, Invitrogen), penicillin-streptomycin (1:100, Invitrogen), B27 supplement without vitamin A (1:50, Invitrogen), and mitogenic growth factors FGF-2 and EGF (Peprotech). For experimental consistency, all experiments were performed in healthy-appearing cultures 24 hours after the second passage which typically occurred on the 8th day in vitro (DIV). After the second passage, 10^4^ NPCs in 100μl of medium was added to the inner 60 wells of a 96-well poly-L-ornithine/laminin coated microplate (BD BioCoat, BD Biosciences, San Jose, CA) using a multichannel pipette (Eppendorf, Westbury, NY), and placed in a humidified cell culture incubator at 37°C with 5% CO_2_ overnight prior to treatment the next morning. The outer wells lining the plate were not used for the experiments because of significant evaporation of the medium over time, especially in experiments > 24 h (i.e., the ‘edge effect’). These wells were filled only with 100μl of medium without the NPCs. Plates were randomly assigned to experiments, and all treatments within an experiment were carried out in the same plate to eliminate the effect of inter-plate variability.

### Oxytocin treatment

We initially selected three concentrations of sOT (10 pM, 100 pM, and 100 nM) for our studies because they closely reflect the typical plasma levels of oxytocin observed during pregnancy.[33] A 100 pM concentration reflects a plasma oxytocin level of approximately 100 pg/mL (molar mass of oxytocin = 1007 g/moL). Because we noted no differences in NPC death, apoptosis, proliferation, and differentiation between the 10 and 100 pM in initial experiments, we eliminated the 10 pM concentration from subsequent experiments to accommodate all treatment conditions (control, 100 pM and 100 nM) within the same experimental plate. Oxytocin (1 mg, Phoenix Pharmaceuticals Inc., Burlingame, CA) was freshly solubilized in 1 mL B27 medium on the day of the experiments (1 mM stock solution) and subsequently diluted to the required concentrations with B27 medium.

### Detection of OXTR

We first investigated if NPCs express OXTRs using both immunocytochemistry and western blotting. For OXTR immunocytochemistry, 5 x 10^4^ NPCs were seeded overnight on Corning® BioCoat™ Poly-D-Lysine/Laminin 12 mm coverslips, permeabilized, incubated overnight with 2μg/ml of mouse anti-nestin (EMD Millipore) and 10μg/ml of goat anti-rat OXTR antibody (Acris Antibodies Inc.) and visualized with species-matched Alexa-Fluor® secondary antibodies. The presence of OXTR was also confirmed with Western blot using the Protein Simple Wes™ automated Western blotting system with 1.5 μg of total protein.[34] Goat anti-OXTR antibody (Acris antibodies) was used at a dilution of 1:10 and human uterus lysate (Abcam, ab44038) was used as positive control.

### Effect of sOT on OXTR

Next, we determined if prolonged treatment with sOT affected the expression of OXTR protein. 5x10^5^ NPCs were seeded overnight in a 6-well poly-L-ornithine laminin coated plate and then were treated with 100 nM sOT for 24h. Immediately after treatment, the NPCs were washed twice with ice-cold PBS, lysed with a protease-phosphatase cocktail, and followed by extraction of total protein. Western blot was performed as described with goat anti-OXTR antibody (Acris; 1:10 dilution) and GAPDH as the loading control.

### NPC viability

Finally, we studied the effect of sOT on NPC viability, proliferation, and differentiation. For viability experiments, NPCs were plated overnight in poly-L-ornithine/laminin coated 96-well plates at a density of 5 x 10^3^ cells/well before treatment with B27 medium with or without 100 pM or 100 nM sOT for either 6 or 24h. Viability was assessed by both propidium iodide (PI) and activated caspase-3 (CC3) immunocytochemistry. PI staining was performed by adding 100μL of 1:100 propidium iodide (2mg/mL stock, Invitrogen) in B27 medium to each well after completion of exposure and allowed to incubate for 5 min prior to fixation with 4% PFA. For CC3 immunocytochemistry, NPCs were fixed at the end of exposure and processed as previously described.[31]

### NPC proliferation

We assessed the effect of sOT exposure and withdrawal on NPC proliferation using a similar experimental paradigm. Proliferation was quantified with the established markers ethynyl deoxyuridine (EdU), an exogenous thymidine analogue that incorporates into the DNA during the S-phase of cell division, and Ki-67, an endogenous marker that labels cells in all phases of the cell cycle except G_0_.[35] For the 6h exposure, EdU (10μM) was added at the same time as the sOT, whereas in the 24h sOT exposure group it was added 6h before cessation of exposure. In the withdrawal experiments, the medium was replaced with proliferation medium (B27 + growth factors) at the end of sOT exposure. Subsequently, EdU (10μM) was added 18h later for the last 6h of the experiment. NPCs were then fixed and processed for Click-iT chemistry (Life Technologies, Carlsbad, CA) as previously described by us.[31, 32] For Ki67 immunocytochemistry, NPCs were fixed and processed at the same time points as for the EdU experiments using a high throughput image analysis system (see below).

### NPC differentiation

To investigate the effect of sOT on differentiation and cell fate selection, NPCs were plated overnight in poly-L-ornithine/laminin coated 96-well plates at a density of 1.5 x 10^3^ cells/well and were then exposed to sOT (100 pM and 100 nM) or vehicle in B27 medium for 24 h. Exposure was terminated by replacing the medium with maintenance B27 medium without growth factors in order to induce spontaneous NPC differentiation. The medium was then replaced every other day for 14 days and on day 14 the cells were fixed and phenotyped as neurons (Neu N), oligodendrocytes (Olig2), or astrocytes (GFAP) with commercially available antibodies. The various antibodies used and their concentrations are listed in **Table 1**. Secondary antibodies were always species-specific, wavelength compatible Alexa-Fluor^®^ antibodies at a final concentration of 10 μg/ml in 3% BSA.

**Table 1:** List of primary antibodies.

Primary Antibody	Target/Event	Reactivity	Host	Concentration/ Dilution	Manufacturer
Anti-Nestin MAB353	NPC	Rat, Mouse	Mouse	2 μg/ml	EMD Millipore
Anti-OXTR AP22376PU-N	Oxytocin Receptor	Rat, Human	Goat	4 μg/ml	Acris Antibodies, Inc
Anti-CC3 9661S	Cleaved-caspase 3	Rat, Mouse, Human, Monkey	Rabbit	1:400	Cell Signalling Technology
Anti-Ki67 Ab 16667	Proliferation	Rat, Mouse, Human, Marmoset	Rabbit	10 μg/ml	Abcam
Anti-Neu N MAB377	Neuron	Rat	Mouse	5 μg/ml	EMD Millipore
Anti-Olig 2 sc-48817	Oligodendrocyte	Rat, Mouse, Human	Rabbit	1:400	Santa Cruz
Anti-GFAP MAB360	Astrocyte	Human, Rat, Mouse, Chicken, Rabbit	Mouse	1:500	EMD Millipore

### Imaging and analysis

Viability, proliferation, and differentiation were assessed in 9 images per well using an automated, unbiased imaging system (IN Cell Analyser 2000, GE Healthcare, Piscataway, NJ).[32] For identification and analysis of EdU, PI and CC3 positive cells, we used a two-step filtering process. In the first step, nestin-negative cells were excluded; in the second, threshold setting was used to determine the number of EdU, PI, and CC3 positive cells. Thus, only nestin-reactive cells were analysed and included in the final analysis. Imaging parameters were set based on the control wells stained with the fluorophore or antibody of interest and the same parameters were used to image all treated wells on the plate (**Table 2**). In these experiments, images were acquired with a 20x objective from 12–18 wells per exposure condition per assay per time point. For cellular phenotyping with specific markers, custom thresholds were set to identify NeuN^+^, GFAP^+^, and Olig2^+^ cells. Imaging and analysis were performed by two separate investigators, but true blinding was not possible because of the necessity to label the exposure condition on the plate to minimize errors.

**Table 2:** Threshold settings for image analysis.

Marker	Target/Event	Parameter	Threshold
PI	cell death	nuclear/cellular intensity ratio	> 2
CC3	apoptosis	nuclear/cellular intensity ratio	> 2.5
EdU	S-phase	nuclear intensity coefficient of variation (CV)	> 0.25
Ki67	proliferation	nuclear/cellular intensity ratio	> 1.5

### Placental transfer of oxytocin

The question whether sOT crosses the placental barrier is not fully resolved because of species differences in placental structure, particularly, the anatomical type of placenta and the thickness of the placental barrier.[36–38] Animal studies, therefore, reveal conflicting results, with studies in sheep showing minimal to no transfer,[39, 40] and those in guinea pigs, baboons, and rats suggesting transplacental transfer of oxytocin. [41–43] Human studies have been conflicting as well, with clinical studies suggesting minimal oxytocin transfer across the placenta,[44] while placental perfusion studies suggesting otherwise.[3] To ensure that our model is internally consistent, we administered varying bolus doses of intravenous sOT (0, 100 mcg/kg, 1 mg/kg) through a 22g tail vein catheter in pregnant Sprague Dawley rats at E20 under brief 2% isoflurane anesthesia. Pooled fetal cardiac blood samples were collected at 15 min after injection with the dam still under isoflurane anesthesia, centrifuged for 3000 rpm for 15 min, and plasma was stored at -80˚C. Samples were C-18 extracted, lyophilized, and assayed for oxytocin in duplicate using a fluorescent enzyme immunoassay kit (#FEK-051-01, Phoenix Pharmaceuticals, Inc.).

### Statistical analysis

We designed our experiments based on previous experience with this *in vitro* system,[31, 32] where a sample size of 3 biologically independent NPC cultures provided at least 80% power to detect a difference between groups at a significance level of 0.05 for a two-sided test. Because of the need to include positive controls for apoptosis and cell death in the same plate, these experiments were conducted on 12 wells/ exposure condition, whereas all other experiments had a minimum sample size of 18 wells/ condition. Western blot data were analysed with Student’s t-test. Experiments with 3 treatment conditions (control, 100 pM, 100 nM) and 2 time points (6h and 24h, or 24h after either a 6 or 24h exposure) were analyzed with 2-way ANOVA followed by post-hoc Bonferroni correction to compare treatments with control group. All other data were analysed using 1-way ANOVA followed by Dunnett’s multiple comparison tests against control exposure if it passed Bartlett’s test for equal variances. Data that did not pass equal variance testing were analysed with Kruskal-Wallis test followed by Dunn’s testing for multiple comparisons. Data are represented as mean ± S.E.M from 3 biological replicates. Data analysis was performed with Prism 5 for MAC OS X software (Graphpad Software, Inc, La Jolla, CA). A two-tailed P value ≤ 0.05 was accorded statistical significance.

## Results

### NPCs express OXTR

**Fig 1:**
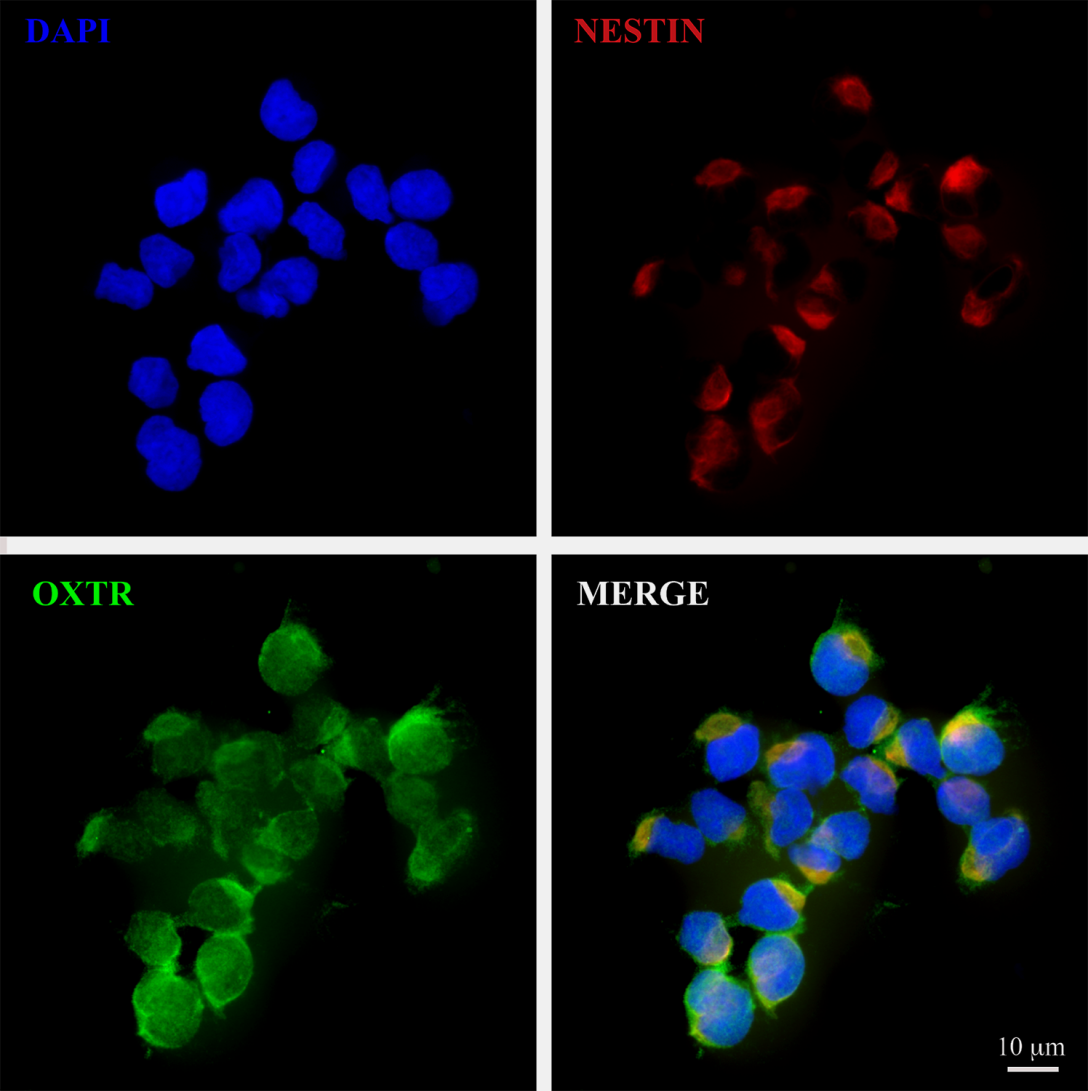
NPCs express OXTR. A 60x photomicrograph showing co-expression of OXTR (green) both in the cytoplasm as well as the plasma membrane of nestin^+^ NPCs (red). Imaging was performed in Olympus confocal FV1000 microscope and processed with Adobe Photoshop. Scale bar as noted.

Our culture conditions yielded approximately 98% pure NPCs, defined by the expression of the neural progenitor cell marker nestin, as reported previously.[32] OXTR was detectable by fluorescence immunocytochemistry (Fig 1) confirming that NPCs express the OXTR.

### Prolonged exposure to sOT downregulates OXTR

**Fig 2:**
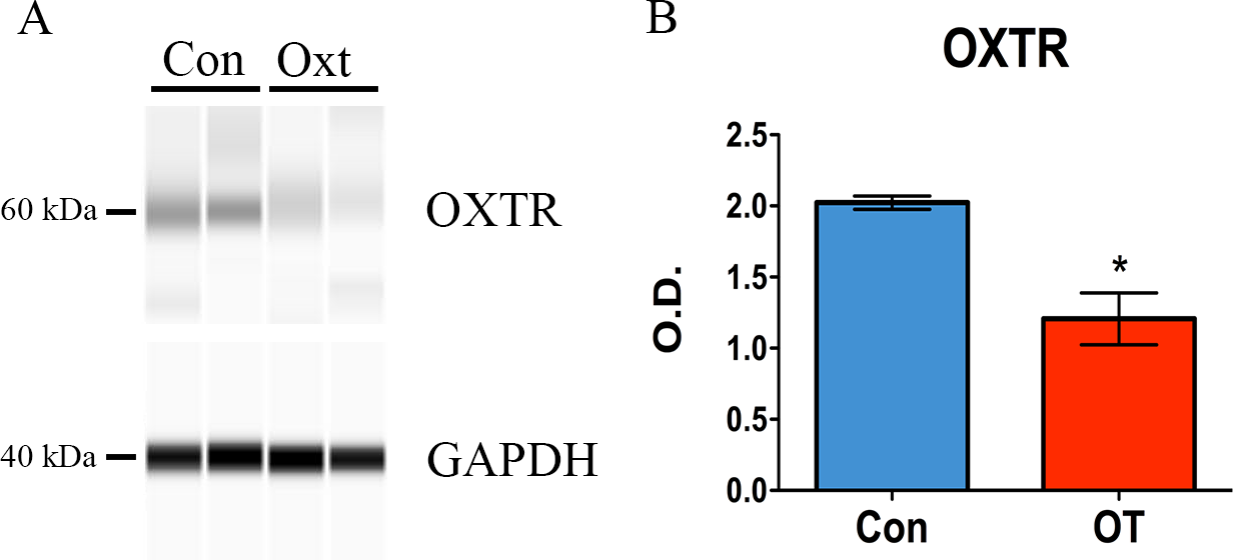
Oxytocin downregulates OXTR in NPCS. 1.5 μg of total protein was analyzed with Protein Simple Wes™ automated Western blotting system. Goat anti-OXTR antibody was used at a dilution of 1:10. Human uterus lysate was used as positive control. Cropped representative pseudo-blots show the expression of OXTR (60 kDa), with GAPDH as the loading control (Fig 2A). Full-length pseudo-blots are presented in Supplementary S1 Fig. Compared to control treatment, 24 h of 100 nM oxytocin significantly downregulated OXTR protein by approximately 45% (*P = 0.01 by Student’s t test) (Fig 2B). Data expressed as mean ± S.E.M from three biologically independent NPC cultures.

Compared to a vehicle control, a 24h exposure to 100 nM sOT downregulated OXTR protein expression in NPCs by approximately 45% as revealed by Western blot (*P = 0.01 by Student’s t test; Fig 2).

### sOT does not affect NPC viability

**Fig 3:**
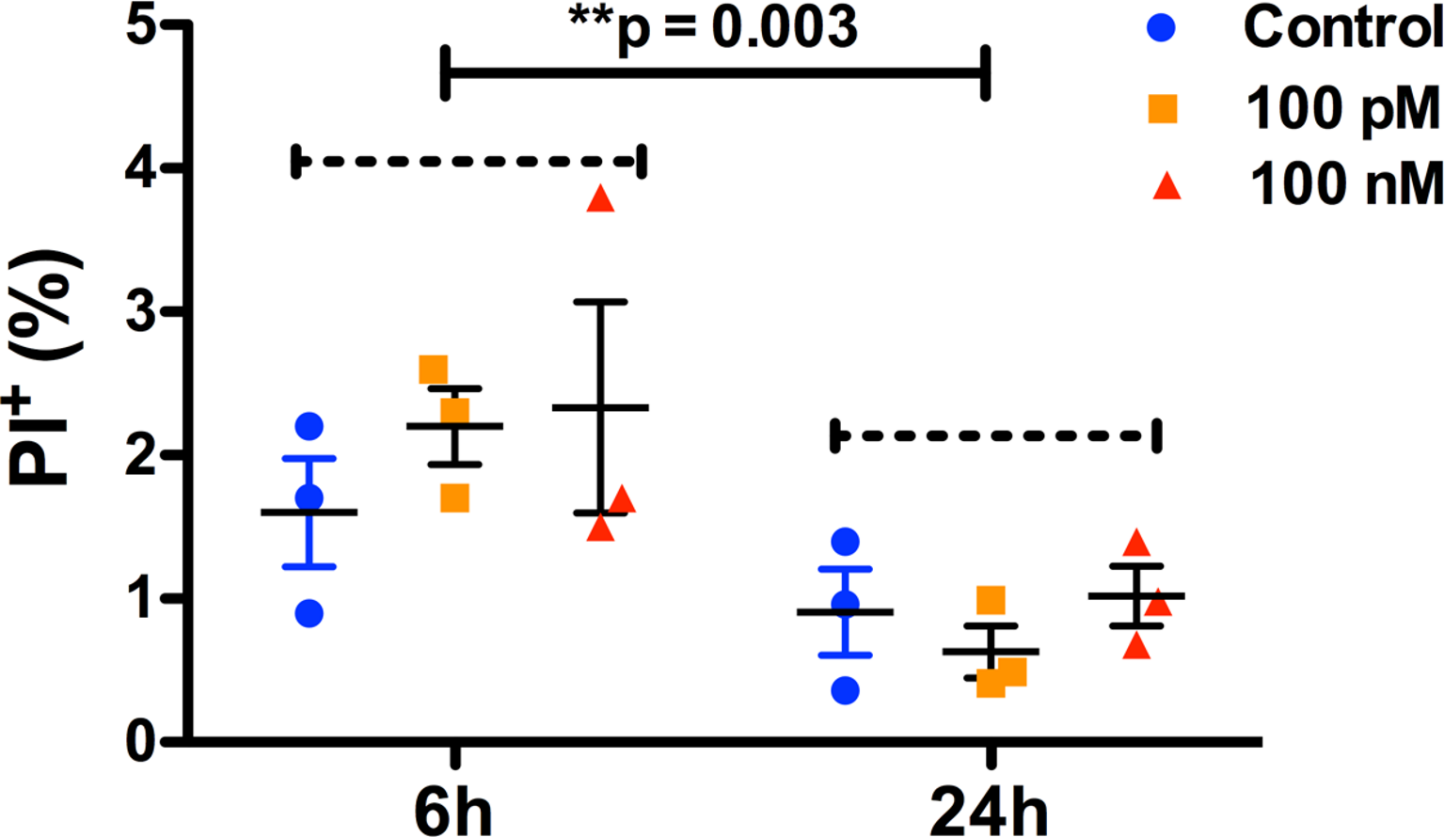
No difference in NPC death at the end of oxytocin treatment. Scatter plots showing the proportion of dead NPCs after treament with either 0, 100 pM, or 100 nM oxytocin for either 6 or 24 h, as noted. NPC death was quantified with PI staining. 2-way ANOVA analysis did not show a difference either with oxytocin treatment (*F* (2,12) = 0.59, p = 0.58, η^2^ = 0.04) or a treatment*time interaction (*F* (2, 12) = 0.66, p = 0.53, η^2^ = 0.04). However, there was a significant effect of time (*F* (1, 12) = 14, *p = 0.003, η^2^ = 0.49) with a lower rate of NPC death at 24h compared to 6h. Data are expressed as mean ± S.E.M from three biologically independent NPC cultures.

**Fig 4:**
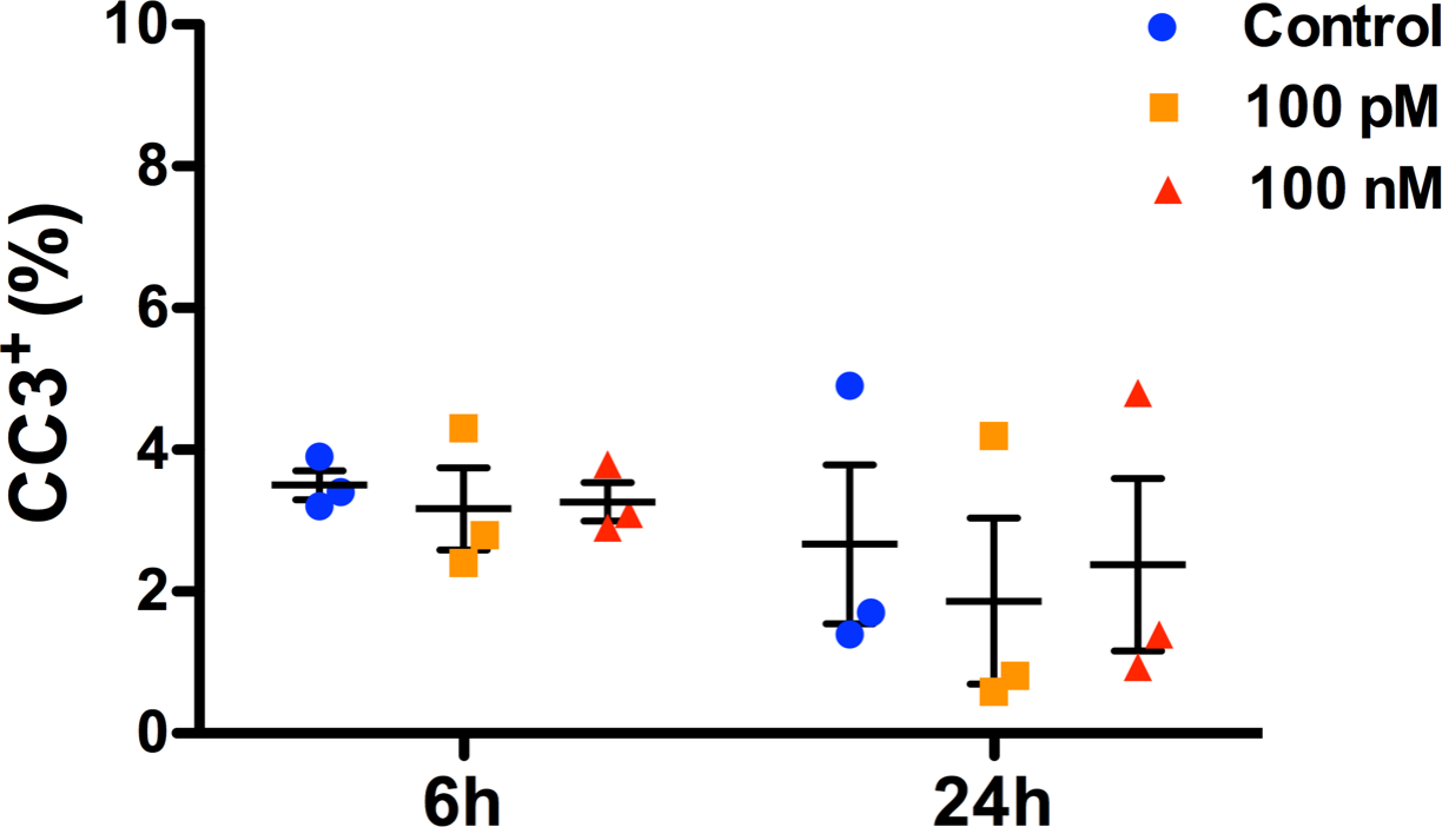
No difference in NPC apoptosis at the end of oxytocin treatment. Scatter plots showing the proportion of apoptotic NPCs after treament with either 0, 100 pM, or 100 nM oxytocin for either 6 or 24 h, as noted. Apoptosis of NPCs was quantified with cleaved caspase-3 immunocytochemistry. 2-way ANOVA analysis did not show a significant difference either with oxytocin treatment (*F* (2,12) = 0.21, p = 0.81, η^2^ = 0.03), time (*F* (1, 12) = 2, p = 0.18, η^2^ = 0.14) or a treatment*time interaction (*F* (2, 12) = 0.043, p = 0.96, η^2^ = 0.006). Data are expressed as mean ± S.E.M from three biologically independent NPC cultures.

Neither 6 nor 24 h exposure to 100 pM or 100 nM sOT had an effect on NPC viability as assessed by either PI (Fig 3) or CC-3 immunocytochemistry (Fig 4). However, the overall proportion of PI^+^ NPCs were significantly lower at the 24 h compared to the 6 h time point (*F* (1, 12) = 14, **p = 0.003, η^2^ = 0.49)

### sOT has minimal effect on NPC proliferation

**Fig 5:**
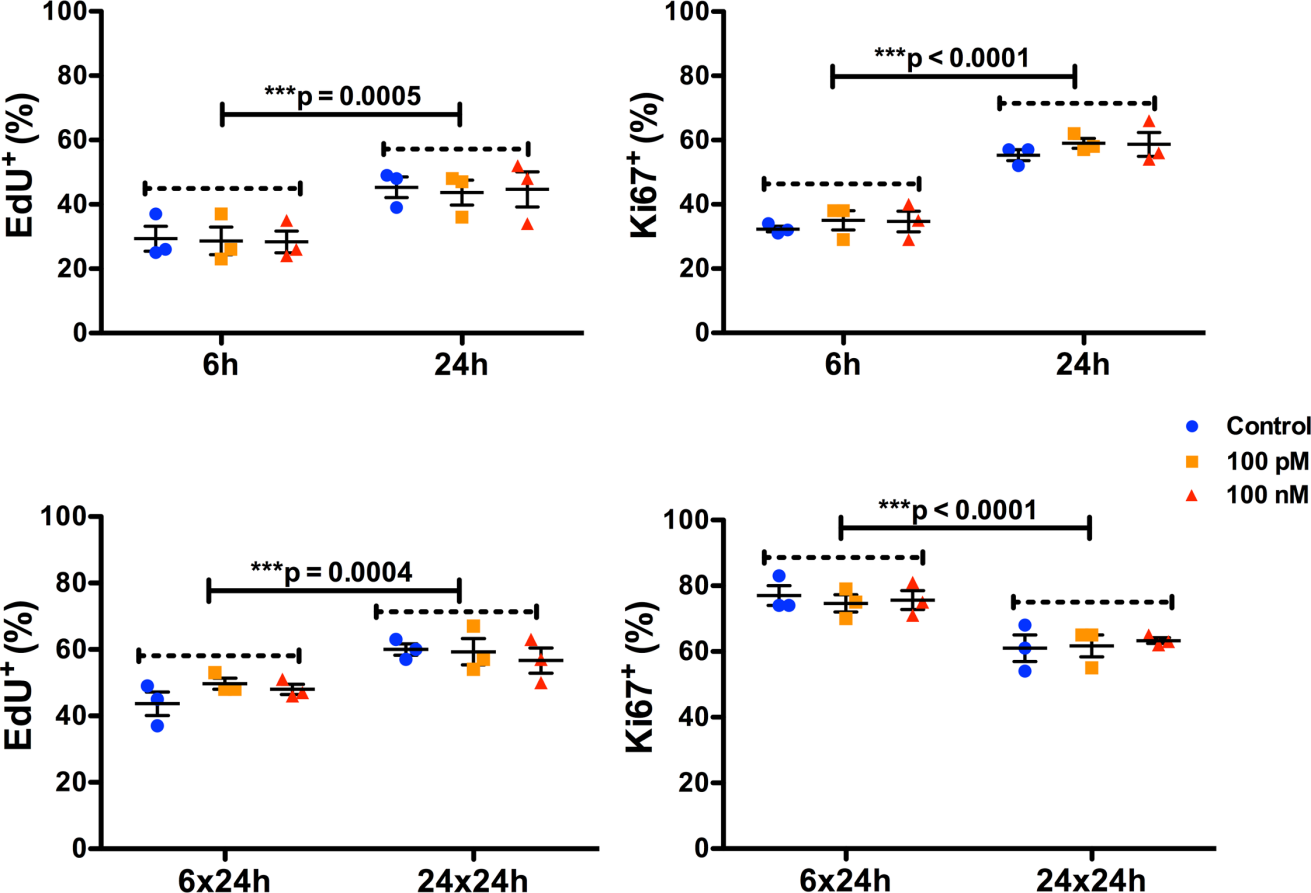
Oxytocin treatment has minimal effect on NPC proliferation. Scatter plots showing the proportion of proliferating NPCs treated with either 100 pM or 100 nM of oxytocin for 6 or 24 h, and after 24 h following removal of oxytocin from the medium. Proliferation was quantified with EdU incorporation and Ki67 immunocytochemistry. There were no differences in NPC proliferation with oxytocin treatment either at 6 or 24 h, though the overall rate of proliferation was significantly higher in the 24 vs the 6 h group for both EdU incorporation and Ki-67 immunoreactivity. There were no significant treatment*time interactions for either EdU incorporation or Ki-67 immunoreactivity. The results were very similar in the oxytocin withdrawal experiments, except that Ki-67 immunoreactivity was significantly lower in the 24 compared to the 6 h group. Data are expressed as mean ± S.E.M from three biologically independent NPC cultures.

**Fig 6:**
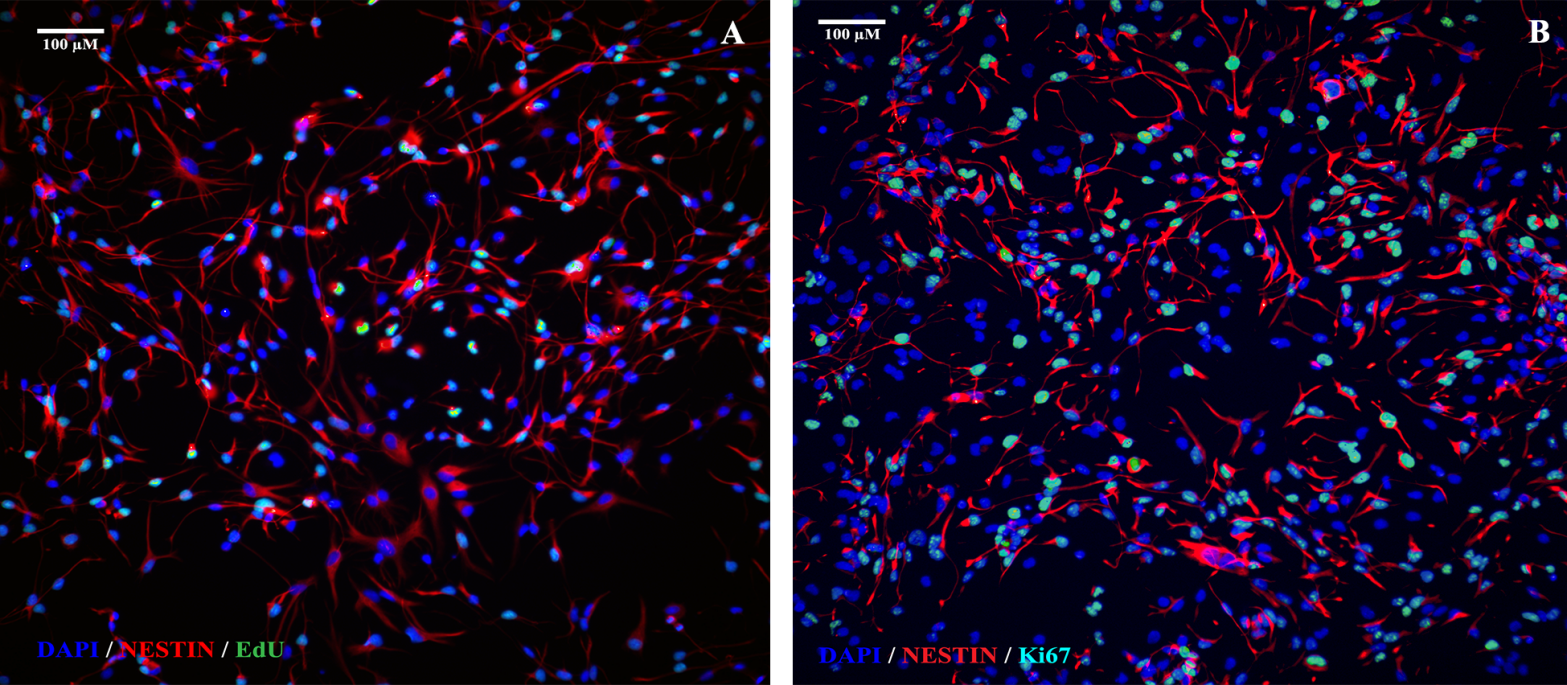
Representative photomicrographs showing EdU and Ki67 immunocytochemistry. 20x images of EdU and Ki67 immunoreactivity from control wells are shown in A and B, respectively. Approximately 35% of NPCs were positive for EdU and approximately 60% were positive for Ki67 immunoreactivity. Scale bar as noted.

**Fig 7:**
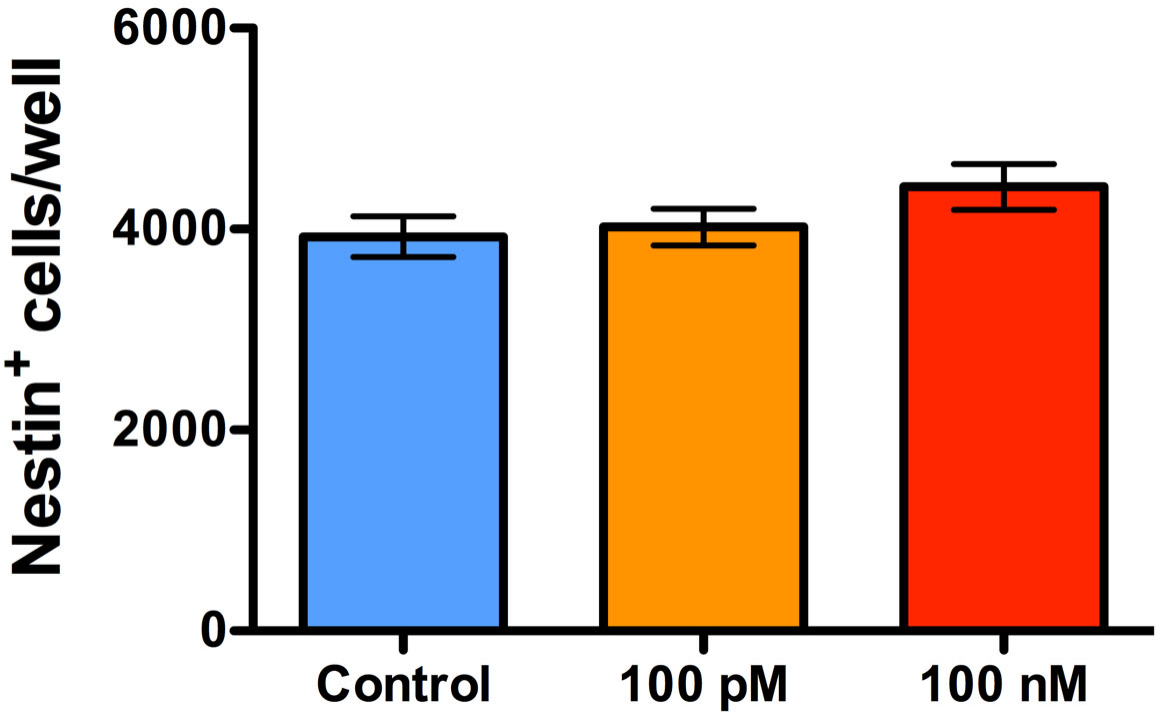
Prolonged treatment with oxytocin does not decrease the neural stem cell pool. A bar graph showing the proportion of NPCs 24 h after treatment with either 100 pM or 100 nM of oxytocin for 24 h. NPCs were phenotyped with nestin immunocytochemistry. There were no differences in the overall proportion of nestin-positive NPCs/well at 24 h after treatment with both concentrations, compared to control treatment. Data are expressed as mean ± S.E.M from three biologically independent NPC cultures.

A 6 h exposure to sOT had no effect on NPC proliferation as assessed by either EdU incorporation (*F* (2, 12) = 0.044, p = 0.96, η^2^ = 0.0008) or Ki-67 immunoreactivity (*F* (2, 12) = 0.94, p = 0.42, η^2^ = 0.013) (Figs 5 & 6). Similarly, a 24 h exposure to sOT had no effect either on NPC proliferation as assessed by either EdU incorporation (*F* (2, 12) = 0.48, p = 0.63, η^2^ = 0.025) or Ki-67 immunoreactivity (*F* (2, 12) = 0.10, p = 0.90, η^2^ = 0.005). However, there was a significant effect of time, with NPC proliferation significantly higher in the 24 vs. 6 h exposure for both EdU incorporation (*F* (1, 12) = 23, p = 0.0005, η^2^ = 0.65) and Ki-67 immunoreactivity (*F* (1, 12) = 130, p < 0.0001, η^2^ = 0.90). There were no significant differences in the treatment*time interaction in either 6 or 24 h exposures to sOT. This pattern continued even 24 h after withdrawal of sOT from the medium, except that Ki-67 immunoreactivity was lower at 24 compared to 6 h (*F* (1, 12) = 33, p < 0.0001, η^2^ = 0.72). No significant differences were observed with EdU incorporation except for a higher rate in the 24x24 group compared to the 6x24 group (*F* (1, 12) = 24, p = 0.0004, η^2^ = 0.61). Neither treatment nor treatment*time interactions achieved statistical significance for EdU incorporation or Ki-67 immunoreactivity. Finally, there was no change in the proportion of nestin positive NPCs 24 h after withdrawal from a 24 h exposure to 100 pM or 100 nM sOT (P = 0.21 vs. control by 1-way ANOVA; Fig 7), indicating that prolonged exposure to sOT does not decrease the NPC pool.

### Prolonged exposure to sOT enhances neuronal but impairs astrocytic and oligodendrocytic differentiation

**Fig 8:**
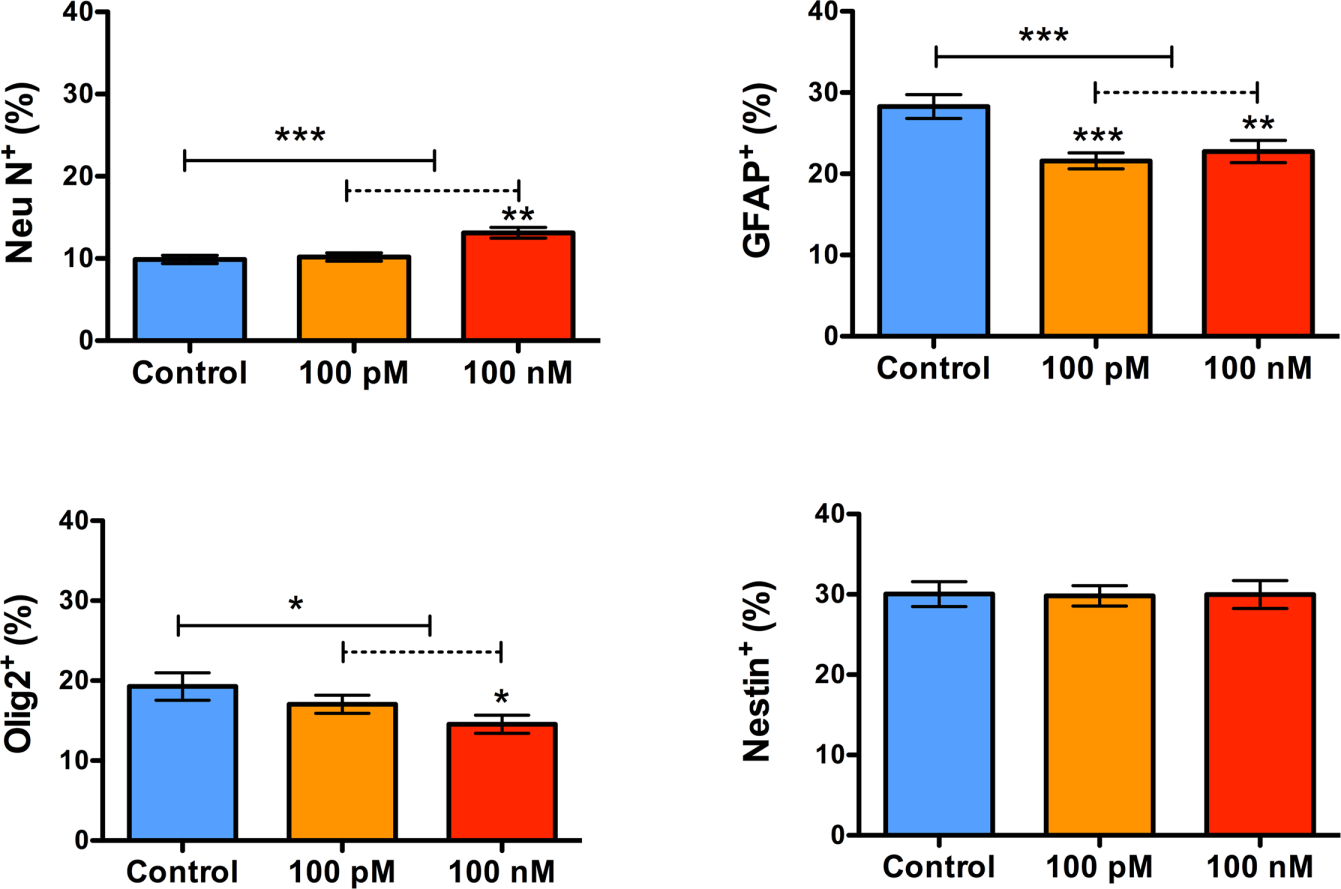
Prolonged exposure to oxytocin enhances neuronal but impairs astrocytic and oligodendrocytic differentiation. Bar graphs showing the proportion of neurons (Neu N), astrocytes (GFAP), oligodendrocytes (Olig2), and nestin^+^ NPCs, two weeks after treatment with 100 nM oxytocin for 24 h followed by mitogen withdrawal. Treatment with oxytocin increased the number of NeuN^+^ neurons (***P = 0.0005 by Kruskal-Wallis test), but decreased the number of both GFAP^+^ astrocytes (***P = 0.0009 by 1-way ANOVA) and Olig2^+^ oligodendrocytes (*P = 0.04 by 1-way ANOVA). These changes were not accompanied by a change in the overall proportion of nestin^+^ NPCs (approximately 30%) (P = 0.97 by Kruskal-Wallis test).

**Fig 9:**
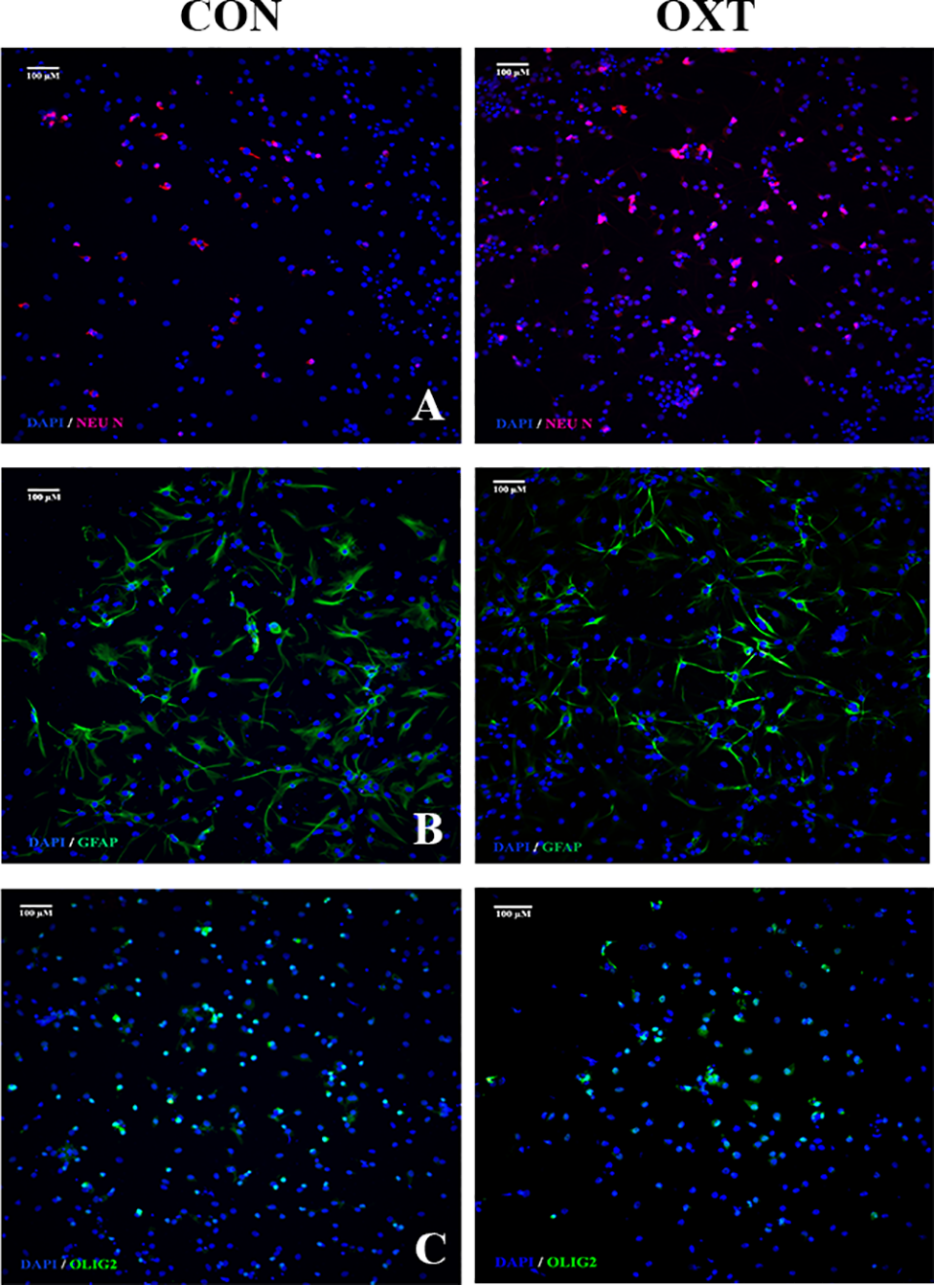
Representative photomicrographs of spontaneously differentiating NPCs after oxytocin exposure (100 nM). A panel showing 10x photomicrographs of neuronal (A), astrocytic (B), and oligodendrocytic (C) differentiation as labeled by Neu N, GFAP, and Olig2, respectively, in control (*left*) and oxytocin (*right*) treated NPCs. Nuclei counterstained with DAPI. Scale bar as noted.

Exposure to sOT for 24 h increased the number of NeuN^+^ neurons (***P = 0.0005 by Kruskal-Wallis test) 2 weeks later but decreased the number of both GFAP^+^ astrocytes (***P = 0.0009 by 1-way ANOVA) and Olig2^+^ oligodendrocytes (*P = 0.04 by 1-way ANOVA) (Fig 8). The proportion of nestin^+^ NPCs (approximately 30%) was unchanged from control (P = 0.97 by Kruskal-Wallis test), indicating that sOT-induced neuronal differentiation is not due to amplification of the progenitor pool. Representative photomicrographs are presented in Fig 9.

### Dose-dependent increase in fetal plasma oxytocin

**Fig 10:**
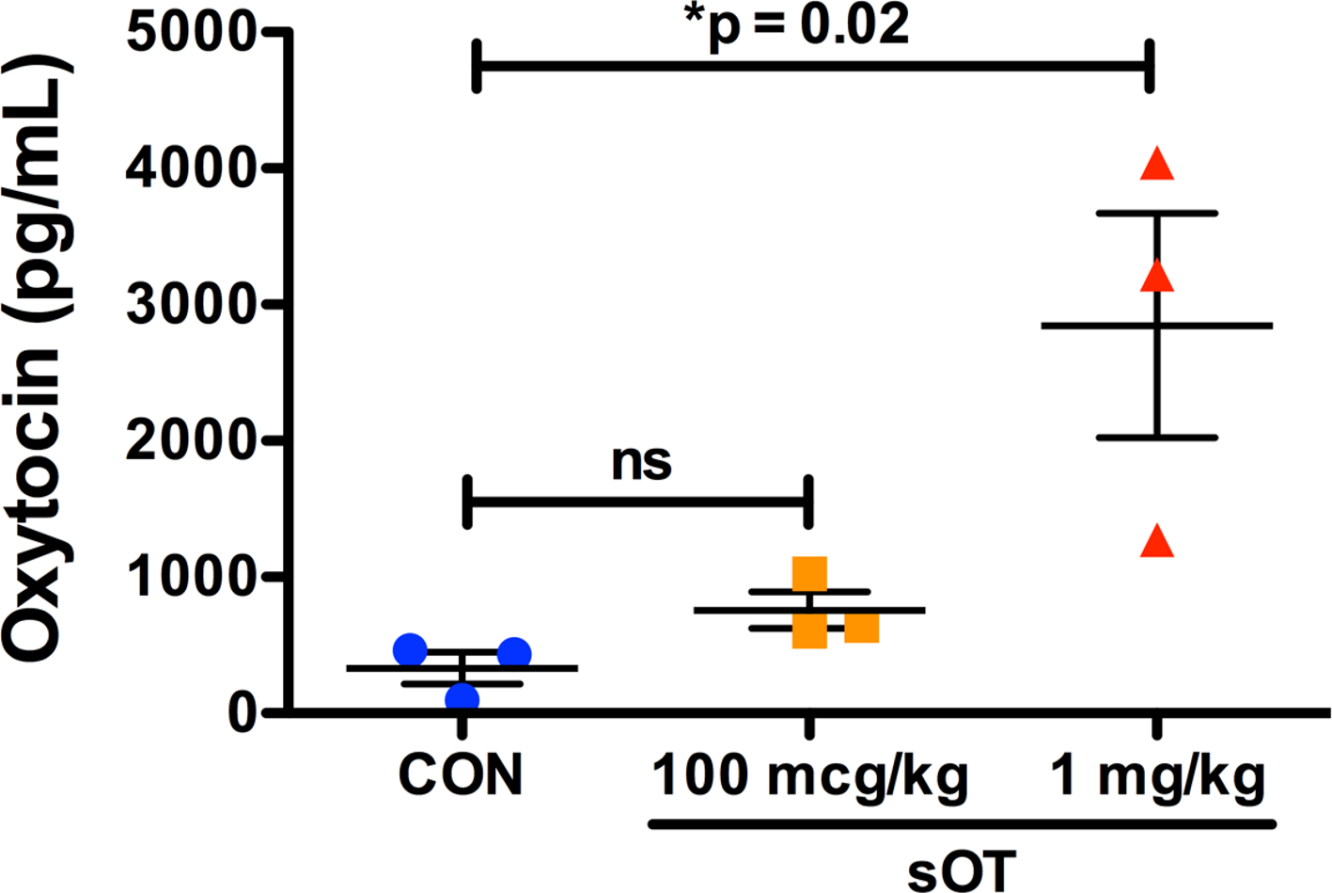
Dose-dependent transfer of sOT across the placenta. Fetal plasma oxytocin level was quantified in pooled fetal cardiac blood samples from dams treated with either saline, 100 mcg/kg, or 1 mg/kg of sOT. Fetal plasma oxytocin increased in the 1 mg/kg dose group (*p = 0.02 by one-way ANOVA; mean ± S.E.M), suggesting that sOT can cross the placenta.

Maternal sOT administration increased fetal plasma oxytocin in a dose-dependent manner, especially at the dose of 1 mg/kg (Fig 10).

## Discussion

Our results demonstrate that neural progenitor cells express the oxytocin receptor and that expression of the receptor is downregulated with prolonged exposure to clinically relevant concentrations of oxytocin. Exposure to oxytocin increased neuronal fate selection and decreased the generation of astrocytes and oligodendrocytes. However, oxytocin had no effect on NPC proliferation and viability. Collectively, these data suggest that exposure to oxytocin could have consequences during neurodevelopment.

OXTRs are present during early development both in neural and non-neural tissues suggesting that oxytocin is intricately involved in modulating facets of early development.[4, 5, 7, 8, 23, 45–49] Here, we show for the first time that NPCs express the OXTR, similar to astrocytes and glial cells in the developing brain.[7] OXTR immunoreactivity was detectable both in the cytoplasm as well as in the periphery of NPCs suggestive for its presence within the plasma membrane. This, however, requires verification and analysis with higher resolution methods. Prolonged exposure to a clinically relevant concentration of sOT decreased OXTR protein expression by almost 45%. This was expected because sOT is known to cause receptor desensitization and downregulation in other tissues such as the uterus.[50–53] We cannot say whether this is an enduring phenomenon, but it raises the possibility that maternally administered sOT may alter the availability and responsiveness of OXTRs to endogenous oxytocinergic signalling in vivo. This could have functional consequences for the fetus because sOT shifts GABA_A_ receptor activity from excitation to inhibition during labor and delivery, an effect that is mediated by the OXTR.[43, 54] In addition, early life manipulation of the OXTR system alters social and sexual behavior in mice and prairie voles.[46, 47, 55–57] Whether this occurs in vivo during maternal administration is not known, but our findings make a case for preclinical studies to investigate neurobehavioral outcomes following prenatal sOT exposure.

The most prominent effect of oxytocin was a change in the cell fate selection of NPCs, with exposure to oxytocin increasing generation of neurons and decreasing production of oligodendrocytes and astrocytes. This is consistent with evidence that oxytocin affects differentiation in other cell types including mesenchymal stem cells, cardiomyocytes, myoepithelial cells, and SH-SY5Ycells, where it promotes neuronal growth.[27, 30, 49, 58] Furthermore, systemic administration of oxytocin enhances differentiation of adipocytes and myoepithelial cells in rodents in vivo.[26, 30] The mechanisms by which oxytocin exerts these effects are unclear, but in the case of NPCs, it may involve oxytocin-driven changes in cellular excitability. Cellular excitation is sufficient to induce neurogenesis.[59] Furthermore, GABAergic excitation is critical for neuronal differentiation,[60] and considering that oxytocin modulates GABAergic excitation,[43, 54] it is not surprising that oxytocin has such a profound effect on NPC differentiation. Our data corroborate these results, albeit for the first time in a neural cell line. The functional significance of increased neuronal and decreased glial cell generation are unknown but increased neuronal density in specific brain regions,[61, 62] and altered white matter connectivity,[63] are both associated with autism spectrum disorders.

Compared to its effect on differentiation, sOT had negligible effect on NPC proliferation. We did not observe a change on NPC proliferation with either 6 or 24 h of sOT treatment. However, 24 h after removal of sOT from the medium, EdU incorporation was higher in the 24 compared to the 6 h treatment group, while Ki-67 immunoreactivity revealed a decrease in the 24 h treatment group. The exact reason for this conflicting result with the two methods is unclear but none of these effects of sOT on NPCs were accompanied by altered cell death or apoptosis. Collectively, we conclude that the major effect of sOT is on NPC differentiation rather than proliferation. Finally, our proof-of-principle experiments show that fetal plasma oxytocin increases in a dose-dependent manner after maternal sOT administration. However, it must be noted that oxytocin is administered as a continuous infusion in clinical practice and our results may not be directly applicable to that setting. Determining whether this increase in fetal plasma oxytocin influences the oxytocin content in the fetal brain is an additional challenge because the fetal brain produces endogenous oxytocin, and commercially available ELISAs do not reliably distinguish between the two. However, there is ample evidence from other animal studies that peripherally administered oxytocin crosses both the mature blood brain barrier (BBB) of the adult,[21, 64, 65] as well as the immature BBB of the developing brain.[54, 66]

Our study has limitations. First, being an in vitro study, caution is required before extrapolating our results to the in vivo situation. Likewise, without a neurodevelopmental endpoint, the biological significance of the changes in cell fate selection is unclear. Second, the CSF concentration of sOT achieved during maternal administration for augmentation of labor is not known. However, we chose concentrations based on CSF levels of oxytocin achieved during pregnancy and labor[67–69] and it is likely that CSF oxytocin levels are even higher when sOT is given exogenously. Third, though EdU is widely used as a proliferative marker, its incorporation reflects ongoing DNA synthesis and, therefore, can also change in conditions unrelated to cell proliferation such as DNA repair and abortive cell cycle re-entry. It is for this reason that we used Ki67, an endogenous marker that captures proliferative cells at all stages of the cell cycle except G_0_, as an independent marker—with similar results at both 6 and 24 h after sOT. Fourth, we cannot rule out the possibility that sOT can affect neurogenesis through modulation of non-OXTR calcium signalling mechanisms.[70, 71] In addition, there is also a potential possibility for interaction between anesthetic agents and sOT especially in the setting of emergent general anesthesia for fetal distress during labor. Finally, we used pharmaceutical grade sOT for our experiments but clinically available oxytocin contains 0.5% chlorobutanol (mol. wt 177.45 g/moL) as preservative. It remains to be seen if this preservative has any direct impact or modulates the effects of sOT on NPCs, but at concentrations < 10 μg/mL chlorobutanol does not appear to have an embryotoxic effect.[72]

## Conclusions

In summary, our in vitro data provide the first evidence that NPCs express the oxytocin receptor, that receptor expression decreases in these cells upon exposure to clinically relevant concentrations of sOT, and that exposing NPCs to sOT generates more neurons but fewer glia. The clinical ramifications of these results are unknown but in vivo studies to determine the morphologic and neurobehavioral consequences of maternal oxytocin therapy on the fetus seem warranted.

## Supporting information

S1 Fig Supplementary figure for western blot. Full-length uncropped pseudo-blots confirming the presence of oxytocin receptor (OXTR) as a 60 kDa band in neural progenitor cells (NPC) and its downregulation upon 24h treatment with 100 nM oxytocin (1A). Because we could not identify GAPDH in uterus lysate, our positive control, we used ß-actin (shown as a 46 kDa band in lanes 2 and 3 in 1B) during our initial experiments to validate the OXTR antibody (lane 1 in 1B). For quantitative experiments comparing untreated vs. oxytocin treated NPCs, we used GAPDH (shown as a 40 kDa band) as our loading control because ß-actin was poorly expressed in NPCs (1C). Here, we used lysates of MCF-7 cells, which are known to express OXTR, as our positive control. All experiments were performed in the Protein Simple Wes™ automated Western blotting system.
(PDF)
