# Supplementary material for: Oxytocin alters cell fate selection of rat neural progenitor cells in vitro
Source: PLoS One. 2018 Jan 18;13(1):e0191160. doi: 10.1371/journal.pone.0191160 (PMC5773179; doi:10.1371/journal.pone.0191160)
Supplement: S1 Fig — Full-length uncropped pseudo-blots confirming the presence of oxytocin receptor (OXTR) as a 60 kDa band in neural progenitor cells (NPC) and its downregulation upon 24h treatment with 100 nM oxytocin (1A). Because we could not identify GAPDH in uterus lysate, our positive control, we used ß-actin (shown as a 46 kDa band in lanes 2 and 3 in 1B) during our initial experiments to validate the OXTR antibody (lane 1 in 1B). For quantitative experiments comparing untreated vs. oxytocin treated NPCs, we used GAPDH (shown as a 40 kDa band) as our loading control because ß-actin was poorly expressed in NPCs (1C). Here, we used lysates of MCF-7 cells, which are known to express OXTR, as our positive control. All experiments were performed in the Protein Simple Wes™ automated Western blotting system. (PDF) [file pone.0191160.s001.pdf]

OXYTOCIN ALTERS CELL FATE SELECTION OF RAT NEURAL PROGENITOR CELLS IN VITRO

Arvind Palanisamy*, Ramaswamy Kannappan†, Audrey Martino‡, Matthew B. Friese‡, Justin D. Boyd§, Gregory Crosby‡, Deborah J. Culley‡

Supplementary Figure 1

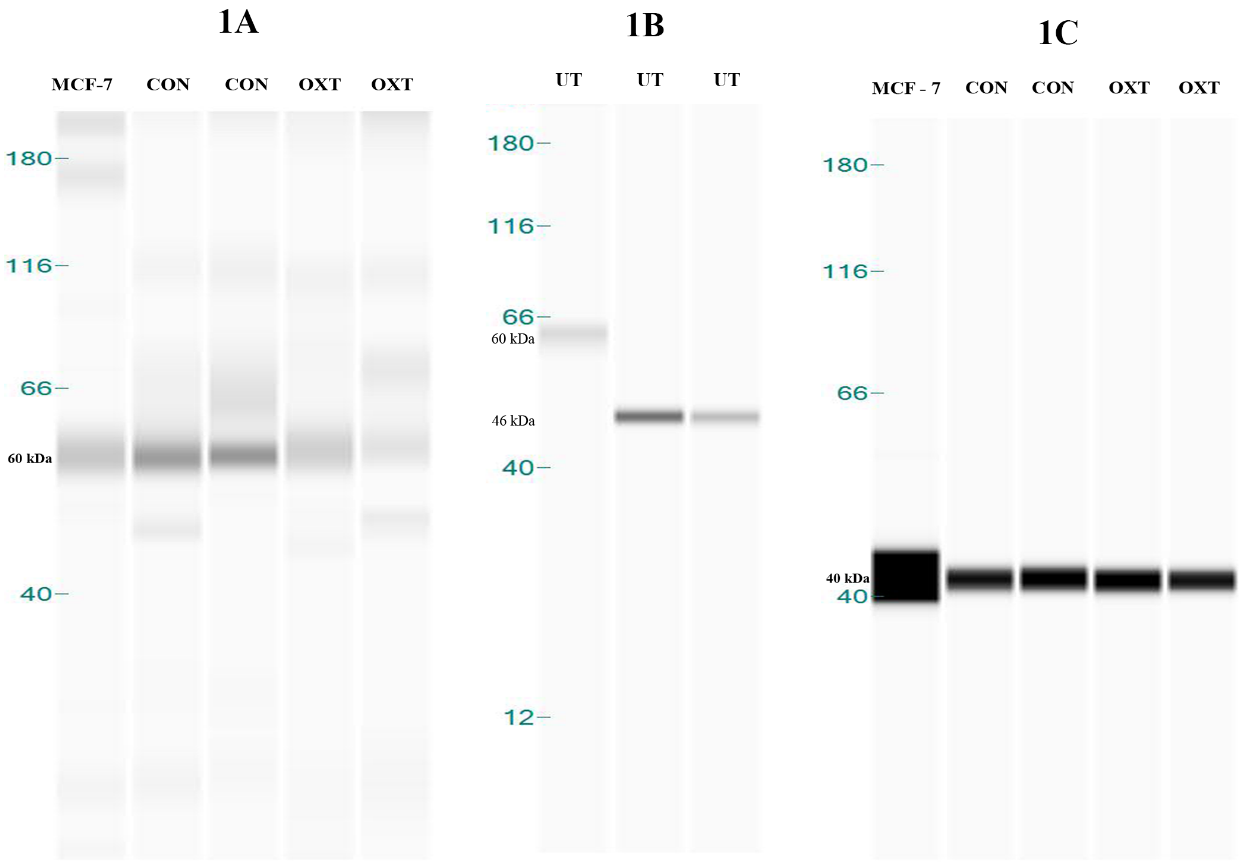

Supplementary Figure 1: Full-length uncropped pseudo-blots confirming the presence of oxytocin receptor (OXTR) as a 60 kDa band in neural progenitor cells (NPC) and its downregulation upon 24h treatment with 100 nM oxytocin (1A). Because we could not identify GAPDH in uterus lysate, our positive control, we used ß-actin (shown as a 46 kDa band in lanes 2 and 3 in 1B) during our initial experiments to validate the OXTR antibody (lane 1 in 1B). For quantitative experiments comparing untreated vs. oxytocin treated NPCs, we used GAPDH (shown as a 40 kDa band) as our loading control because ß-actin was poorly expressed in NPCs (1C). Here, we used lysates of MCF-7 cells, which are known to express OXTR, as our positive control. All experiments were performed in the Protein Simple Wes™ automated Western blotting system.
